# Multimodal phenotypic labelling using drug‐induced sleep endoscopy, awake nasendoscopy and computational fluid dynamics for the prediction of mandibular advancement device treatment outcome: a prospective study

**DOI:** 10.1111/jsr.13673

**Published:** 2022-06-22

**Authors:** Karlien Van den Bossche, Sara Op de Beeck, Marijke Dieltjens, Annelies E. Verbruggen, Anneclaire V. Vroegop, Johan A. Verbraecken, Paul H. Van de Heyning, Marc J. Braem, Olivier M. Vanderveken

**Affiliations:** ^1^ Faculty of Medicine and health Sciences University of Antwerp Wilrijk Belgium; ^2^ ENT, Head and Neck Surgery Antwerp University Hospital Edegem Belgium; ^3^ Multidisciplinary Sleep Disorders Centre Antwerp University Hospital Edegem Belgium; ^4^ Department of Pulmonary Medicine Antwerp University Hospital Edegem Belgium

**Keywords:** obstructive sleep apnea (OSA), oral appliances, personalised medicine, treatments

## Abstract

Mandibular advancement device (MAD) treatment outcome for obstructive sleep apnea (OSA) is variable and patient dependent. A global, clinically applicable predictive model is lacking. Our aim was to combine characteristics obtained during drug‐induced sleep endoscopy (DISE), awake nasendoscopy, and computed tomography scan‐based computational fluid dynamic (CFD) measurements in one multifactorial model, to explain MAD treatment outcome. A total of 100 patients with OSA were prospectively recruited and treated with a MAD at fixed 75% protrusion. In all, 72 underwent CFD analysis, DISE, and awake nasendoscopy at baseline in a blinded fashion and completed a 3‐month follow‐up polysomnography with a MAD. Treatment response was defined as a reduction in the apnea–hypopnea index (AHI) of ≥50% and deterioration as an increase of ≥10% during MAD treatment. To cope with missing data, multiple imputation with predictive mean matching was used. Multivariate logistic regression, adjusting for body mass index and baseline AHI, was used to combine all potential predictor variables. The strongest impact concerning odds ratios (ORs) was present for complete concentric palatal collapse (CCCp) during DISE on deterioration (OR 28.88, 95% confidence interval [CI] 1.18–704.35; *p* = 0.0391), followed by a C‐shape versus an oval shape of the soft palate during wakefulness (OR 8.54, 95% CI 1.09–67.23; *p* = 0.0416) and tongue base collapse during DISE on response (OR 3.29, 95% CI 1.02–10.64; *p* = 0.0464). Both logistic regression models exhibited excellent and fair predictive accuracy. Our findings suggest DISE to be the most robust examination associated with MAD treatment outcome, with tongue base collapse as a predictor for successful MAD treatment and CCCp as an adverse DISE phenotype.

## INTRODUCTION

Obstructive sleep apnea (OSA) is a common syndrome with a worldwide prevalence of almost 1 billion people in the 30–69 years age range (Benjafield et al., 2019). This sleep‐related breathing disorder is characterised by recurrent events of partial or complete collapse of the upper airway, lasting ≥10 s during sleep, leading to a reduction in respiratory flow (Gottlieb & Punjabi, 2020). Currently, the most commonly used standard in assessing sleep apnea severity is the apnea–hypopnea index (AHI), which presents the amount of partial (hypopnea) and complete (apnea) collapses of the upper airway per hour of sleep.

Continuous positive airway pressure (CPAP) is currently considered the standard therapy but has a limited compliance rate (Guralnick et al., 2017). In this regard, an alternative non‐invasive treatment option for patients with OSA is oral appliance therapy, the most of which are custom‐made, titratable mandibular advancement devices (MADs). A MAD is worn intra‐orally at night and acts by protruding the mandible, resulting in an opening of the upper airway and an increase in volume of the upper airway (Chan et al., 2010). In this respect, multiple studies have already proven the efficacy of MADs, but not in all patients: therapy success may range from 47.7% to 75.0% (Gjerde et al., 2016; Kim et al., 2014).

The variable and patient‐dependent MAD treatment response emphasises the need for careful patient selection. Selection for MAD treatment today relies on baseline patient characteristics, anthropometrics, and drug‐induced sleep endoscopy (DISE) findings with or without the use of a simulation bite (Chen et al., 2020; Op de Beeck et al., 2019; Vroegop et al., 2020). However, a global, clinically applicable predictive model is lacking.

### Prediction of MAD treatment outcome

#### Baseline Parameters

In previous studies, various treatment response factors for MAD therapy are described: younger age, female gender, supine‐dependent OSA, lower body mass index (BMI), lower AHI, retracted maxilla and mandible, narrower airway, and shorter soft palate than non‐responders (Chen et al., 2020; Pahkala et al., 2020; Sutherland et al., 2015). Nevertheless, these parameters only show a weak association in predicting MAD treatment efficacy.

#### Computational fluid dynamics

Functional imaging can be used to investigate OSA and the mechanism of action of MADs on the upper airway morphology, with the use of computed tomography (CT) scans on three‐dimensional (3D), computer‐aided design, coupled with computational fluid dynamics (CFD). In several studies here, MADs have been proven to act by enlarging the upper airway volume and the minimal cross‐sectional area in order to prevent upper airway collapse during sleep, stating that a decrease in upper airway resistance and an increase in upper airway volume are correlated with an objective clinical improvement of OSA severity (De Backer et al., 2007; Vos et al., 2007). Moreover, a smaller minimal cross‐sectional area is a marker for higher OSA severity (Vos et al., 2007). In this regard, recent analysis has shown that MADs may act by increasing the total upper airway volume, predominantly due to an increase in velopharyngeal volume (Van Gaver et al., 2021). Furthermore, particularly in responders to MAD treatment, Van Gaver et al. have found a significant increase in total upper airway volume, emphasising that the efficacy of a MAD is associated with a larger increase in upper airway volume. On the other hand, the absence of an increase in velopharyngeal volume seems to be associated with deterioration. Additionally, previous studies have also found an association between treatment response of a MAD and total upper airway volume with a predominant increase in velopharyngeal volume (Chan et al., 2010; Song et al., 2019). These markers may be used in evaluating treatment outcome in patients with OSA. Therefore, the combination of imaging techniques and CFD may play a role in future MAD‐personalised patient selection.

#### Drug‐induced sleep endoscopy

The observed site(s) and pattern(s) of upper airway collapse during DISE are proven to play a major role in personalised treatment selection of non‐CPAP therapy for patients with OSA (Op de Beeck et al., 2019; Vanderveken et al., 2013; Vroegop et al., 2020). Accordingly, a complete concentric collapse at the level of the palate (CCCp) has been shown to be associated with a less favourable surgical outcome for upper airway stimulation therapy (Vanderveken et al., 2013). Then, according to another recent study, CCCp was shown to be associated with a negative MAD treatment outcome (Op de Beeck et al., 2019). That study also showed an association of complete oropharyngeal collapse with an adverse effect on MAD treatment and a higher success rate of a MAD with the presence of tongue base collapse during baseline DISE (Op de Beeck et al., 2019).

#### Awake nasendoscopy

Awake nasendoscopy with Müller's manoeuvre is an Ear, Nose, Throat (ENT) investigation, commonly used in the clinical examination of patients diagnosed with OSA. Müller's manoeuvre here is defined as a forced inspiratory effort against a closed airway, where the examiner endoscopically observes the narrowing of the pharyngeal walls at the retrolingual and retropalatal level. However, various studies show that Müller's manoeuvre has a number of inconsistencies (Soares et al., 2013; Zerpa Zerpa et al., 2015). Nevertheless, Van de Perck et al. demonstrate an alternative awake nasendoscopic evaluation method during tidal breathing, without the need of manoeuvres carried out by patients (Van de Perck, Vroegop, et al., 2020b). A significant correlation has been found between DISE and the following endoscopic features evaluated during tidal breathing on awake nasendoscopy: complete palatal collapse with the position of the soft palate, oropharyngeal collapse with crowding of the oropharynx, complete tongue base collapse with a posteriorly located tongue base, and epiglottis collapse with the modified Cormack–Lehane scale (Van de Perck, Vroegop, et al., 2020b). Furthermore, recent analyses have shown the correlation of two baseline nasendoscopic features during tidal breathing with deterioration under MAD treatment, being oropharyngeal crowding and a posterior location of the soft palate (Van de Perck, Op de Beeck, et al., 2020a).

#### Study aim

Multiple studies report several predictors for oral appliance therapy outcome, which have recently been reviewed (Okuno et al., 2016), although thorough validation is lacking. With this, a recent study of Sutherland et al. has aimed to derive a prediction model based on multiple awake assessments, including facial photography, spirometry, and nasendoscopy (Sutherland et al., 2018). However, no significant added value of these awake assessments has been found compared to the use of only clinical baseline characteristics in the prediction of MAD treatment outcome. Therefore, a persistent need to find a robust clinical applicable prediction model combining several predictors is highlighted.

Accordingly, the aim of this study was to combine findings derived from DISE, awake nasendoscopy and CT scan‐based CFD in one model to explain MAD treatment outcome.

#### Ethical considerations

The present data were prospectively obtained from the Agentschap voor Innovatie door Wetenschap en Technologie (IWT) database of the Predicting Therapeutic Outcome of Mandibular Advancement Device Treatment in Obstructive Sleep Apnea (PROMAD) trial (identifier NCT01532050 on clinicaltrials.gov) (Verbruggen et al., 2016). The ethics committee at the Antwerp University Hospital and the University of Antwerp approved the study. All patients have given a written informed consent prior to participation in the PROMAD cohort study.

## METHODS

**FIGURE 1:**
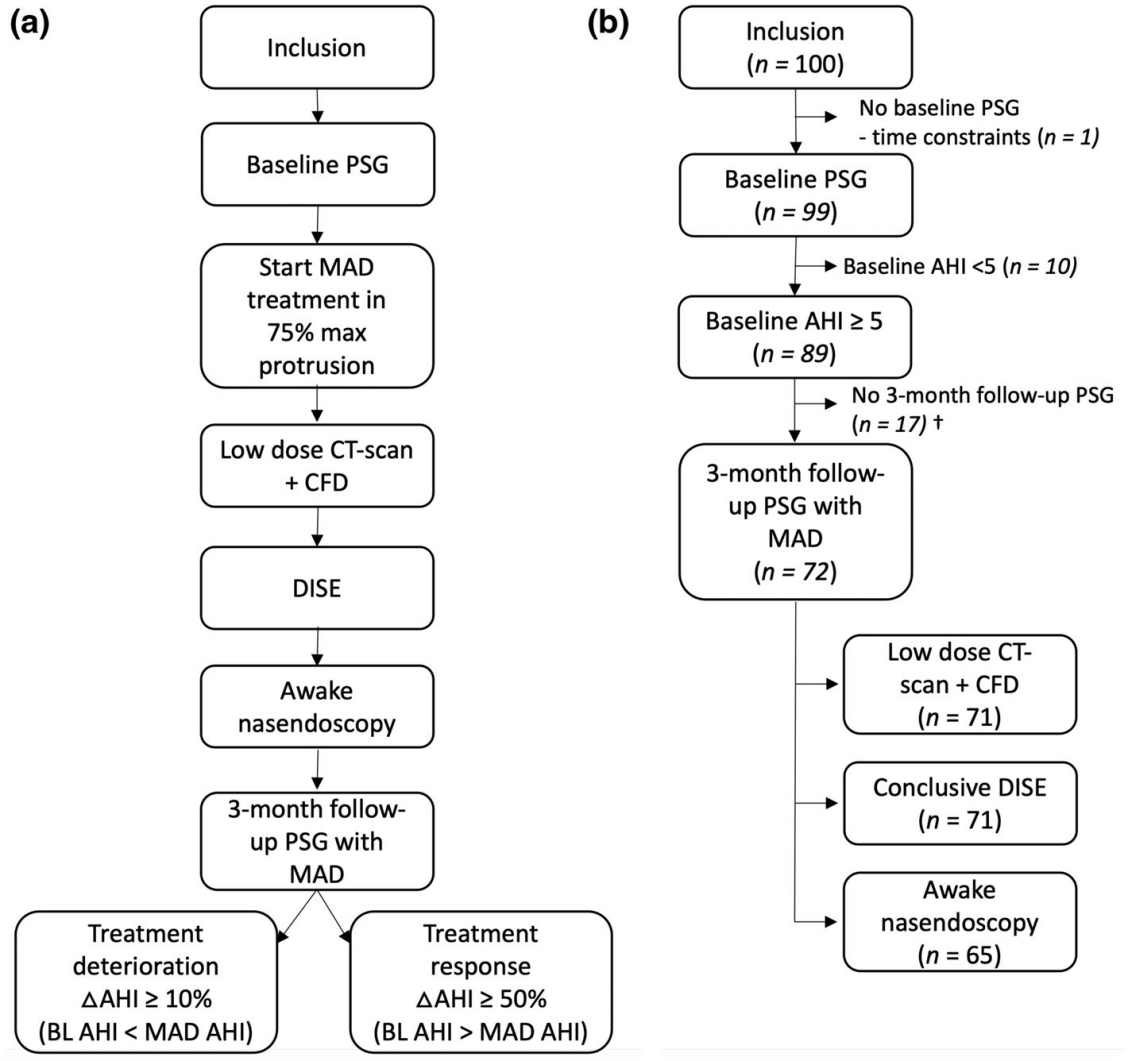
Study flow chart (a) and patient flow (b). †Reasons for dropout: time constraints (seven patients), lost to follow‐up despite several reminders (three), expenses (two), insufficient reduction of complaints with MAD (two), OSA resolution after weight loss (one), excessive gag reflex with MAD (one), and moving abroad (one). Abbreviations: AHI, apnea–hypopnea index; BL, baseline; CFD, computational fluid dynamics; CT, computed tomography; DISE, drug‐induced sleep endoscopy; MAD, mandibular advancement device; OSA, obstructive sleep apnea; PSG, polysomnography

This study protocol was published previously by Verbruggen et al (Figure 1a) (Verbruggen et al., 2016). At first, patients were screened and underwent an extensive clinical examination by an ENT and dental sleep specialist. Temporomandibular joint issues were evaluated anamnestically, through palpation and with a functional assessment of opening or closing of the mouth and movements of deduction of the lower jaw. Subsequently, an objective baseline evaluation on a standard full‐night polysomnography (PSG) by assessing the AHI was made to verify the eligibility criteria (Table 1). Afterwards, oral appliance therapy was initiated using a titratable, custom‐made, duoblock MAD (Respident Butterfly MAD, Orthodontics Clinics NV, Antwerp, Belgium) in 75% of the individualised maximal mandibular protrusion. Each patient's maximum protrusion capacity was measured three times and averaged, using a proprietary gauge bite fork. Measurements were made according to the trajectory of the centric relation position to maximal protrusion. At 3 months after initiation of the MAD, a second PSG was performed to determine the follow‐up AHI. The AHI and other PSG variables were scored by a sleep laboratory technician according to the American Academy of Sleep Medicine (AASM) criteria (Iber et al., 2007). Subsequently, treatment outcome was measured by the difference in baseline AHI and AHI after 3 months of therapy. Deterioration was primarily expressed by an increase in AHI percentage of ≥10% from baseline. Treatment response was defined as a reduction of ≥50% in the AHI with MAD compared to baseline PSG. After initiating MAD treatment, all patients underwent the following three investigations at baseline: a low‐dose CT scan of the head and neck region with CFD analysis 1 month after the start of MAD treatment, a DISE between 1 and 3 months after start, and an awake evaluation using nasendoscopy the day of the 3‐month follow‐up PSG. Furthermore, the investigators and patients remained blinded during the data collection.

**TABLE 1:** Eligibility criteria

**Inclusion criteria**	Age ≥18 years
	Body mass index (BMI) ≤35 kg/m^2^
	OSA as defined by the American Academy of Sleep Medicine task force (Iber et al., 2007)
	Diagnostic criteria: (A + B + D or C + D)
	A. Anamnesis (at least one of the following criteria)
	1 Unwanted sleepiness and/or fatigue in the daytime, unrefreshing sleep, or insomnia
	2 Nocturnal arousals with breathing stops, gasping
	3 Snoring or breathing stops while sleeping, determined by the bed partner
	B. PSG: AHI ≥5 events/h of sleep and AHI <50 events/h of sleep
	C. PSG: AHI ≥15 events/h of sleep and AHI <50 events/h of sleep
	D. The condition cannot be explained by another sleep disorder, internal or neurological disorder, medication, or drug use
**Exclusion criteria**	Absolute dental contraindications:
	‐ Functional restrictions of the temporomandibular joint
	‐ Insufficient dentition with pathological aspects
	‐ Insufficient retention for MAD use
	Other sleep disorders (e.g. parasomnias)
	Previous invasive upper airway surgery for sleep‐disordered breathing
	Genetic disorders with craniofacial and/or upper airway anomalies
	Use of benzodiazepine(s) and/or antidepressant(s)
	Prior history of psychiatric disease (including alcohol abuse)
	Known history of fibromyalgia or chronic fatigue syndrome
	Unwilling to participate and/or to give informed consent

Abbreviations: AHI, apnea–hypopnea index; BMI, body mass index; MAD, mandibular advancement device; OSA, Obstructive sleep apnea; PSG, polysomnography.

### Computational fluid dynamics

During the awake baseline low‐radiation‐dose CT with CFD, patients were placed in a supine position and were asked to hold their breath at the end of a normal inspiration. Based on the scanned areas starting at the nasopharynx down to the larynx, 3D computer‐aided design models were reconstructed using Mimics software (Materialise, Leuven, Belgium). These models were subsequently transferred into a computational grid by FluidDa NV (Kontich, Belgium). The upper airway volume was determined and expressed as the effective upper airway volume in which air flows through, excluding leakage into the mouth. The total volume and the volume of the three individual sections of the pharynx were measured: velopharynx, oropharynx, and hypopharynx. Additional anatomical parameters, such as the minimal cross‐sectional area and the upper airway resistance were calculated.

### Drug‐induced sleep endoscopy

**FIGURE 2:**
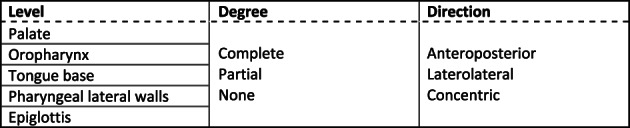
Standard scoring system for DISE. Rows represent the site of upper airway collapse; columns represent level, degree, and direction. Abbreviations: DISE, drug‐induced sleep endoscopy

A DISE was performed in a semi‐dark and silent operating theatre with the patient lying in a supine position. The investigation was performed by an experienced ENT surgeon and scored by a board of four experienced ENT surgeons. Natural sleep was mimicked by administering sedative drugs, induction of sleep was obtained by an intravenous bolus administration of midazolam (1.5 mg) and remained with a target‐controlled infusion of propofol (2.0–3.0 μg/ml). A flexible fiberoptic nasopharyngoscope (Olympus END‐GP, diameter 3.7 mm, Olympus Europe GmbH, Hamburg, Germany) was used and inserted intranasally to inspect the upper airway. Collapse degree (none, partial, or complete), direction (anteroposterior, concentric, or lateral) and level were scored according to a standardised scoring system (Figure 2). The following upper airway levels were examined: soft palate, oropharynx (region at the level of the tonsils), tongue base, epiglottis, and hypopharyngeal lateral walls (region below tongue base).

### Awake nasendoscopy

Patients underwent an awake endoscopic investigation, performed using a flexible fiberoptic nasopharyngoscope (Olympus END‐G, diameter 3.7 mm, Olympus Europe GmbH) by an experienced ENT surgeon. Observations were re‐scored based on the video recordings by a second experienced ENT surgeon. The upper airway was evaluated during tidal breathing in the upright position. During tidal breathing, the same five upper airway levels as during DISE were evaluated based on qualitative upper airway features (Figure 2) (Van de Perck, Vroegop, et al., 2020b). The soft palate was divided in three categorical shapes: the oval shape (anterior position), the C‐shape (prominent uvula), and the dumbbell shape (overall narrowing of the velopharynx due to a posterior location of the soft palate). Oropharyngeal crowding was defined as the presence of large palatine tonsils or the occurrence of prominent pharyngeal arches provoking partial obscuration or compression of the tongue base. The lingual tonsils were scored according to the Friedman grading system (Friedman et al., 2015). The position of the tongue base was categorised depending on the visibility of the valleculae: completely, partially, or not visible and the fourth category consisted of a compression of the epiglottis and/or a posteriorly located tongue base. The epiglottic shape was assessed as a normal, flat, or curved. Lastly, the modified Cormarck–Lehane scale was used to describe the hypopharynx: complete or partial visibility of the vocal cords, visibility of the arytenoids but not of the vocal cords, and no visibility of the glottis (Torre et al., 2018).

### Statistical analyses

All data were analysed using SPSS® Statistics, version 27.0 (IBM Corp.). Descriptive demographic, clinical and PSG parameters were displayed as mean (± standard deviation [SD]) for normally distributed values, medians (25th–75th percentile) for not normally distributed values, or in number of participants or percentages. The paired *t* test for normally distributed values or the Wilcoxon signed‐rank test for not normally distributed values were used to compare changes in baseline and follow‐up variables with MAD treatment. Baseline clinical, PSG, and airway parameters were compared between different MAD treatment outcomes using the Mann–Whitney *U* test, the unpaired *t* test, or the Fisher's exact test for categorical variables.

To deal with missing data, multiple imputation with predictive mean matching was used. The following variables were considered as predictors: gender, age, baseline and maximal mandibular protrusion, AHI, supine AHI, non‐supine AHI, CFD parameters, DISE parameters, and awake nasendoscopy parameters. The pooled data of 10 imputation sets were subsequently compared. Two simple logistic regression models (enter method), explaining MAD treatment response and MAD treatment deterioration were built for DISE, awake nasendoscopic, and CFD observations, with and without correction for BMI and baseline AHI.

Furthermore, all potential predictor variables were combined in separate multivariate logistic regression models (enter method) according to MAD outcome, adjusting for BMI and baseline AHI. Potential predictor variables considered were: DISE (CCCp and tongue base collapse), awake nasendoscopy (soft palatal position and oropharyngeal crowding) and CFD (total upper airway volume and velopharyngeal volume) parameters. Partial and complete tongue base collapse were pooled.

Accuracy was assessed using diagnostic statistics (sensitivity, specificity, positive [PPV] and negative predictive value [NPV]) and using receiver operating characteristic (ROC) analysis. The optimal probability cut‐off was assessed using the Youden's index. Statistical significance was set at *p* < 0.05 with two‐sided *p* values.

## RESULTS

A total of 100 patients with OSA (83% male; mean [SD] age 47.6 [10.0] years; mean [SD] BMI 26.9 [3.3] kg/m^2^; mean [SD] AHI 21.0 [11.2] events/h sleep) were prospectively included for this study from a specialised ENT department in a tertiary care centre (Antwerp University Hospital, Antwerp, Belgium) and underwent a new baseline PSG (Figure 1b). One participant did not undergo this baseline PSG leaving 99 patients. OSA severity was graded as mild (5≤ AHI <15 events/h), moderate (15≤ AHI <30 events/h), or severe (AHI ≥30 events/h). In all, 10 patients who did not have OSA based on this new baseline PSG were excluded (AHI <5 events /h) leaving 89 eligible patients. Follow‐up PSG with MAD was not completed in 17 subjects with 72 patients in the final dataset. In one of the confirmed OSA patients with a follow‐up PSG, a low‐dose CT scan was not performed. All patients underwent DISE, but observations were inconclusive in one patient due to agitation, resulting in 71 with baseline and 3‐month follow‐up PSG and conclusive DISE observations. Moreover, 7 patients did not undergo awake nasendoscopy, resulting in 65 patients. After multiple imputation, a total dataset of 72 patients could be obtained.

### Evolution of clinical characteristics

A significant improvement was seen in clinical characteristics at the 3‐month follow‐up compared to baseline (Table 2). The median (interquartile range [IQR]) AHI (*p* < 0.0001) improved from 15.6 (10.4–23.5) to 9.0 (4.3–16.0) events/h, minimal oxygen saturation (*p =* 0.0020) improved from 86.9% (83.3%–90.0%) to 89.0% (85.8%–91.0%), and the Oxygen Desaturation Index (ODI) (*p* < 0.0001) decreased from 4.2 (2.2–11.3) to 2.0 (0.6–5.0) events/h. Excessive daytime sleepiness as measured with the Epworth Sleepiness Scale (ESS) decreased from a median (IQR) score of 9/24 (5–12) to 6/24 (3–10) (*p* < 0.0001). In terms of treatment outcome, deterioration was seen in 11 patients (15.3%) and response was seen in 33 patients (45.8%). No significant differences were found in baseline clinical characteristics between responders and non‐responders, and between deteriorating and non‐deteriorating patients (Table 3).

**TABLE 2:** Clinical characteristics of the study population at baseline and 3‐month follow‐up

	Baseline PSG (*n* = 72)	3‐month follow‐up PSG (*n* = 72)	*p*
AHI, events/h	15.6 (10.4–23.5)	9.0 (4.3–16.0)	**<0.0001**
AHI supine, events/h	35.3 (18.2–53.4)	12.7 (3.8–30.3)	**<0.0001**
AHI non‐supine, events/h	8.9 (3.9–16.8)	5.0 (2.9–10.6)	**0.0097**
Mean SaO_2_, %	95.3 (94.1–96.1)	95.3 (94.2–96.0)	0.2512
Minimal SaO_2,_ %	86.9 (83.3–90.0)	89.0 (85.8–91.0)	**0.0020**
ODI, events/h	4.2 (2.2–11.3)	2.0 (0.6–5.0)	**<0.0001**
ESS (0–24)	9.0 (5.0–12.0)	6.0 (3.0–10.0)	**<0.0001**
VAS (0–10)	7.0 (5.0–8.0)	6.0 (4.0–9.0)	0.2024
BMI, kg/m^2^	27.8 (3.3)	28.1 (3.3)	**0.0109**

Abbreviations: AHI, apnea–hypopnea index; BMI, body mass index; ESS, Epworth Sleepiness Scale; IQR, interquartile range; ODI, Oxygen Desaturation Index; VAS, visual analogue scale for snoring; PSG, polysomnography.

*Note*: Values are presented as median (IQR [quartile 1–quartile 3]) for non‐normally distributed data or mean (SD) for normally distributed data. All parameters were compared using the Wilcoxon signed‐rank test. BMI was compared using a paired *t* test. AHI was scored according to the American Academy of Sleep Medicine 1999 criteria (3% oxygen desaturation or an arousal). ODI was calculated as dips of ≥3% over the total time in bed. Significant values (*p* < 0.05) are shown in bold.

**TABLE 3:** Clinical and demographic characteristics of the study population at baseline according to mandibular advancement device treatment outcome

	Total sample (*n* = 72)	Response		Deterioration	
		Yes (*n* = 33)	No (*n* = 39)	Yes (*n* = 11)	No (*n* = 61)
Gender, male/female, *n*	61/11	26/7	35/4	9/2	52/9
Age, years	48.3 (10.0)	48.2 (9.6)	48.4 (10.3)	48.1 (9.0)	48.3 (10.2)
BMI, kg/m^2^	27.8 (3.3)	28.1 (3.0)	27.5 (3.5)	27.5 (3.7)	27.8 (3.2)
AHI, events/h	15.4 (10.4–23.5)	17.3 (10.6–25.3)	14.6 (10.4–23.3)	12.8 (7.9–23.2)	17.3 (10.5–24.0)
VAS (0–10)	7.0 (5.0–8.0)	7.0 (5.0–8.5)	6.0 (4.0–8.0)	8.0 (6.0–9.0)	7.0 (5.0–7.8)
ESS (0–24)	9.0 (5.0–12.0)	10.0 (4.0–15.0)	9.0 (6.0–10.0)	9.0 (5.0–11.0)	9.0 (5.0–12.0)
Baseline protrusion, mm	12.5 (2.0)	12.0 (11.6–14.1)	12.6 (11.7–14.0)	13.5 (12.7–14.0)	12.0 (11.5–14.0)
Maximal protrusion, mm	12.1 (2.8)	11.7 (10.0–13.3)	12.2 (10.3–14.7)	11.3 (9.0–12.4)	12.0 (10.3–14.7)
ODI, events/h	4.2 (2.2–11.3)	4.0 (2.1–10.4)	4.4 (2.3–11.3)	3.2 (1.5–11.3)	4.6 (2.3–11.3)
Mean SaO2, %	95.3 (94.1–96.1)	94.9 (94.0–95.9)	95.3 (94.1–96.2)	95.3 (94.1–96.7)	95.1 (94.1–96.1)
Minimal SaO2, %	86.9 (83.3–90.0)	87.0 (81.5–90.0)	86.4 (84.3–90.0)	88.0 (85.0–90.0)	86.0 (83.0–90.0)

Abbreviations: AHI, apnea–hypopnea index; BMI, body mass index; ESS, Epworth Sleepiness Scale; IQR, interquartile range; MAD, mandibular advancement device; ODI, Oxygen Desaturation Index; VAS, visual analogue scale for snoring.

*Note*: Values are presented as median (IQR [quartile 1–quartile 3]) for non‐normally distributed data; mean (SD) for normally distributed data; or frequencies. All parameters were compared using the Mann–Whitney *U* test. BMI and age were compared using an unpaired sample *t* test. Gender was compared using a Fisher's exact test (two‐sided). AHI was scored according to the American Academy of Sleep Medicine 1999 criteria (3% oxygen desaturation or an arousal). ODI was calculated as dips of ≥3% over the total time in bed. No significant values (*p* < 0.05) were found.

### Comparison of airway parameters according to treatment outcome

Between responders and non‐responders, a significant difference was present during DISE for tongue base collapse (65.6% in responders and 41.0% in non‐responders; *p* = 0.0404) and for palatal collapse (87.5% in responders and 100% in non‐responders; *p =* 0.0240) (Table 4). There were no significant differences in CFD and awake nasendoscopy parameters regarding response or no response.

**TABLE 4:** Airway parameters in responders and non‐responders for mandibular advancement device treatment at baseline

	Response (*n* = 33)	No response (*n* = 39)	*p*
DISE collapse			
Tongue base, *n* (%)	21 (65.6)	16 (41.0)	**0.0404**
CCCp, *n* (%)	5 (15.6)	11 (28.2)	0.2100
Complete laterolateral oropharynx, *n* (%)	1 (3.1)	6 (15.4)	0.0869
Palate, *n* (%)	28 (87.5)	39 (100)	**0.0240**
Oropharynx, *n* (%)	7 (21.9)	15 (38.5)	0.1354
Hypopharyngeal lateral walls, *n* (%)	8 (25.0)	8 (21.6)	0.7420
Epiglottis, *n* (%)	7 (21.9)	6 (15.8)	0.5172
Multilevel collapse, *n* (%)	26 (81.3)	28 (71.8)	0.3563
Awake nasendoscopy during tidal breathing			
Position soft palate, *n* (%)			0.6208
‐ Oval shape	22 (73.3)	24 (68.6)	0.6763
‐ C‐shape	6 (20.0)	7 (20.0)	1.0000
‐ Dumbbell shape	2 (6.7)	4 (11.4)	0.5118
Oropharyngeal crowding, *n* (%)	2 (6.7)	7 (20.0)	0.1237
CFD, median (IQR)			
Total upper airway volume, cm^3^	8.3 (5.7–11.5)	9.3 (5.4–15.7)	0.3049
Velopharyngeal volume, cm^3^	1.9 (0.4–3.6)	2.1 (0.6–5.3)	0.3746
Oropharyngeal volume, cm^3^	3.4 (1.8–4.2)	3.3 (1.9–4.7)	0.6530
Hypopharyngeal volume, cm^3^	2.7 (1.6–5.0)	3.2 (2.3–5.8)	0.1774
Upper airway resistance, Pa/L	0.11 (0.06–0.15)	0.09 (0.06–0.30)	0.9120
Upper airway resistance based radius, mm	2.3 (0.0–2.7)	2.0 (0.0–2.7)	0.8195
Minimal cross‐sectional area, mm	0.25 (0.00–0.58)	0.17 (0.00–0.60)	0.7564

Abbreviations: CCCp, complete concentric collapse at the level of the palate; IQR, interquartile range; MAD, mandibular advancement device; CFD, computational fluid dynamics; DISE, drug‐induced sleep endoscopy.

*Note*: Values are presented as median (IQR [quartile 1–quartile 3]) for non‐normally distributed data or frequencies. All CFD parameters were compared using the Mann–Whitney *U* test. Endoscopic collapse patterns were compared using a Fisher's exact test (two‐sided).

No significant differences at baseline were found between deteriorating and non‐deteriorating patients regarding CFD parameters (upper airway volume, upper airway resistance, and minimal cross‐sectional area) (Table 5). However, significant results were present for the following DISE parameters: CCCp (*p =* 0.0494), and complete laterolateral oropharyngeal collapse (*p* = 0.0364). Moreover, a significant difference was found in awake nasendoscopy parameters during tidal breathing between deteriorating and non‐deteriorating patients with a higher percentage of no deterioration in patients with an oval shaped soft palate (78.2%; *p =* 0.0022) and a higher percentage of deterioration in a C‐shaped soft palate (50.0%; *p =* 0.0105). A significantly higher percentage in oropharyngeal crowding was seen in deteriorating patients (40.0%; *p* = 0.0098) than in non‐deteriorating patients (9.1%).

**TABLE 5:** Airway parameters in deterioration and no deterioration for mandibular advancement device treatment at baseline

	Deterioration (*n* = 11)	No deterioration (*n* = 61)	*p*
DISE collapse			
Tongue base, *n* (%)	7 (63.6)	30 (50.0)	0.4086
CCCp, *n* (%)	5 (45.5)	11 (18.3)	**0.0494**
Complete laterolateral oropharynx, *n* (%)	3 (27.3)	4 (6.7)	**0.0364**
Palate, *n* (%)	11 (100.0)	56 (93.3)	0.3814
Oropharynx, *n* (%)	4 (36.4)	18 (30.0)	0.6770
Hypopharyngeal lateral walls, *n* (%)	4 (36.4)	12 (20.7)	0.2622
Epiglottis, *n* (%)	1 (9.1)	12 (20.3)	0.3818
Multilevel collapse, *n* (%)	10 (90.9)	44 (73.3)	0.2125
Awake nasendoscopy during tidal breathing			
Position soft palate, *n* (%)			**0.0029**
‐ Oval shape	3 (30.0)	43 (78.2)	**0.0022**
‐ C‐shape	5 (50.0)	8 (14.5)	**0.0105**
‐ Dumbbell shape	2 (20.0)	4 (7.3)	0.2044
Oropharyngeal crowding, *n* (%)	4 (40.0)	5 (9.1)	**0.0098**
CFD, median (IQR)			
Total upper airway volume, cm^3^	8.0 (4.2–16.5)	8.8 (5.6–12.4)	0.6795
Velopharyngeal volume, cm^3^	2.3 (0.2–6.2)	2.0 (0.5–3.8)	0.8487
Oropharyngeal volume, cm^3^	3.1 (1.8–4.6)	3.4 (2.0–4.5)	0.4745
Hypopharyngeal volume, cm^3^	3.1 (1.1–5.1)	3.0 (1.9–5.4)	0.7506
Upper airway resistance, Pa/L	0.12 (0.05–0.29)	0.11 (0.06–0.23)	0.9138
Upper airway resistance based radius, mm	2.2 (0.0–2.7)	2.1 (0.0–2.7)	0.7654
Minimal cross‐sectional area, mm	0.23 (0.00–0.67)	0.21 (0.00–0.58)	0.7777

Abbreviations: CCCp, complete concentric collapse at the level of the palate; IQR, interquartile range; MAD, mandibular advancement device; CFD, computational fluid dynamics; DISE, drug‐induced sleep endoscopy.

*Note*: Values are presented as median (IQR [quartile 1–quartile 3]) for non‐normally distributed data or frequencies. All CFD parameters were compared using the Mann–Whitney *U* test. Endoscopic collapse patterns were compared using a Fisher's exact test (two‐sided). Significant values (*p* < 0.05) are shown in bold.

### Simple logistic regression

**FIGURE 3:**
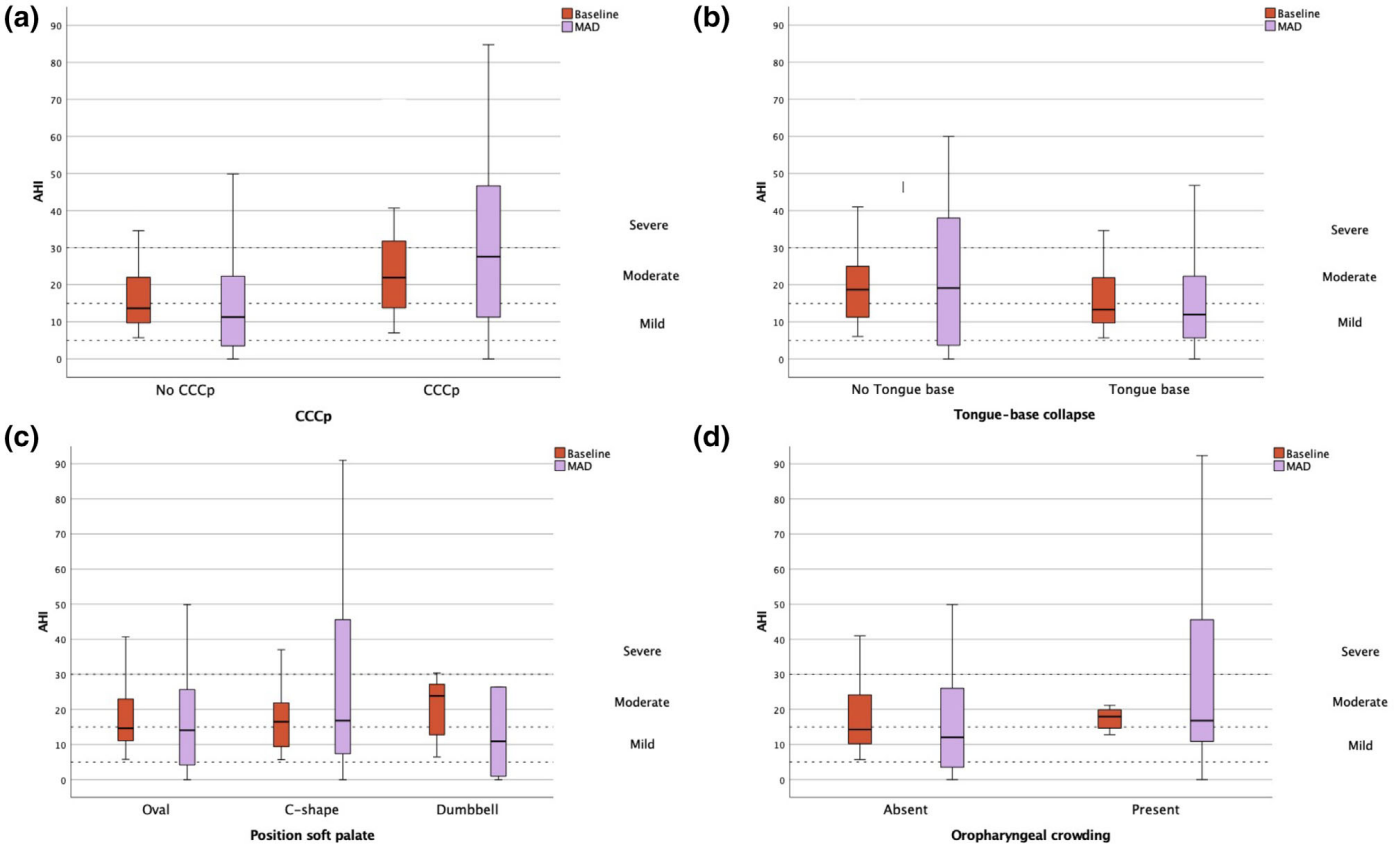
Change in AHI between baseline and MAD use for each categorical potential predictor variable. Change in AHI for all patients without (left) and with (right) tongue base collapse, CCCp, according to the position of the soft palate (oval shape, C‐shape, and dumbbell shape) and in the absence (left) or presence (right) of oropharyngeal crowding. Outliers were omitted in these graphical representations. Abbreviations: AHI, apnea–hypopnea index; CCCp, complete concentric collapse at the level of the palate; MAD, mandibular advancement device.

Based on previous literature (Chan et al., 2010; Op de Beeck et al., 2019; Song et al., 2019; Van de Perck, Op de Beeck, et al., 2020a; Van Gaver et al., 2021), potential predictor variables considered were DISE (CCCp and tongue base collapse) (Figure 3a,b), awake nasendoscopy (soft palatal position and oropharyngeal crowding) (Figure 3c,d), and CFD (total upper airway volume and velopharyngeal volume). In the imputed dataset, a significant relationship was found using simple logistic regression between response and tongue base collapse during DISE with an odds ratio [OR] of 2.69 (95% confidence interval [CI] 1.02–7.10; *p* = 0.0457) (Table 6). This relationship remained significant after correcting for baseline AHI and BMI (*p* = 0.0237; OR 3.43, 95% CI 1.18–9.98). Moreover, similar analysis using simple logistic regression showed a significant relationship between deterioration and CCCp after correction for baseline AHI and BMI (*p* = 0.0269; OR 5.63, 95% CI 1.22–26.03). Concerning awake nasendoscopy, a C‐shaped soft palate and oropharyngeal crowding were associated with deterioration with an OR of 8.31 (95% CI 1.67–41.45; *p =* 0.0098) and an OR of 4.95 (95% CI 1.04–23.60; *p =* 0.0449) with preserved significance after correction for baseline AHI and BMI (OR: 8.27 and OR: 5.12, respectively). There was no relationship between MAD outcome and baseline CFD variables. Correlation tests between the different predictor variables were performed, with only a weak relationship between these variables.

**TABLE 6:** Logistic regression according to mandibular advancement device outcome

	Response				Deterioration			
	Simple logistic regression		+ AHI + BMI		Simple logistic regression		+ AHI + BMI	
	*p*	OR (95% CI)	*p*	OR (95% CI)	*p*	OR (95% CI)	*p*	OR (95% CI)
DISE collapse								
Tongue base	**0.0457**	2.69 (1.02–7.10)	**0.0237**	3.43 (1.18–9.98)	0.4092	1.75 (0.46–6.61)	0.4934	1.62 (0.41–6.44)
CCCp	0.2451	0.50 (0.15–1.62)	0.1555	0.39 (0.10–1.44)	0.0623	3.63 (0.94–14.07)	**0.0269**	5.63 (1.22–26.03)
Complete laterolateral oropharynx	0.2032	0.26 (0.03–2.05)	0.1950	0.25 (0.03–2.03)	0.0697	4.63 (0.88–24.21)	0.0663	5.04 (0.90–28.36)
Palate	0.9992	0.00	0.9992	0.00	0.9992	3134 E10^5^	0.9992	2915 E10^5^
Oropharynx	0.1609	0.47 (0.16–1.35)	0.1439	0.45 (0.15–1.31)	0.6918	1.31 (0.34–5.05)	0.6392	1.39 (0.35–5.48)
Hypopharyngeal lateral walls	0.8338	1.13 (0.37–3.39)	0.9047	1.07 (0.35–3.29)	0.3443	1.94 (0.49–7.63)	0.3003	2.10 (0.52–8.56)
Epiglottis	0.5423	1.44 (0.45–4.67)	0.4775	1.54 (0.47–5.10)	0.3410	0.35 (0.04–3.02)	0.3126	0.33 (0.04–2.86)
Multilevel	0.4098	1.60 (0.52–4.96)	0.3237	1.82 (0.55–6.00)	0.2288	3.71 (0.44–31.31)	0.2697	3.36 (0.39–18.87)
Awake nasendoscopy during tidal breathing								
Soft palate position								
‐ C‐shape (vs. oval shape)	0.8264	0.87 (0.26–2.94)	0.8449	0.89 (0.26–3.01)	**0.0098**	8.31 (1.67–41.45)	**0.0110**	8.27 (1.62–42.13
‐ Dumbbell shape (vs. oval shape)	0.6340	0.67 (0.12–3.57)	0.6580	0.68 (0.12–3.77)	0.1067	5.31 (0.70–40.49)	0.1047	5.98 (0.69–51.87)
Oropharyngeal crowding	0.1132	0.27 (0.05–1.36)	0.1157	0.27 (0.05–1.38)	**0.0449**	4.95 (1.04–23.60)	**0.0470**	5.12 (1.02–25.68)
CFD								
Total upper airway volume	0.0845	1.00 (1.00–1.00)	0.0883	1.00 (1.00–1.00)	0.7982	1.00 (1.00–1.00)	0.8033	1.00 (1.00–1.00)
Velopharyngeal volume	0.2313	1.00 (1.00–1.00)	0.2841	1.00 (1.00–1.00)	0.4669	1.00 (1.00–1.00)	0.5810	1.00 (1.00–1.00)
Minimal cross‐sectional area	0.3559	1.00 (0.98–1.01)	0.4450	1.00 (0.98–1.01)	0.6112	1.00 (0.99–1.02)	0.7874	1.00 (0.99–1.02)
Upper airway resistance	0.7782	0.85 (0.26–2.73)	0.6445	0.73 (0.20–2.73)	0.5110	0.46 (0.04–4.82)	0.6068	0.53 (0.04–6.28)
Upper airway resistance based radius	0.8214	0.96 (0.66–1.39)	0.9086	0.98 (0.67–1.43)	0.6833	1.11 (0.66–1.87)	0.8142	1.07 (0.63–1.79)

Abbreviations: AHI, apnea–hypopnea index; BMI, body mass index; CCCp, complete concentric collapse at the level of the palate; CFD, computational fluid dynamics; CI, confidence interval; DISE, drug‐induced sleep endoscopy; MAD, mandibular advancement device; OR, odds ratio.

*Note*: Data measured on imputed database.

### Multivariate logistic regression

**FIGURE 4:**
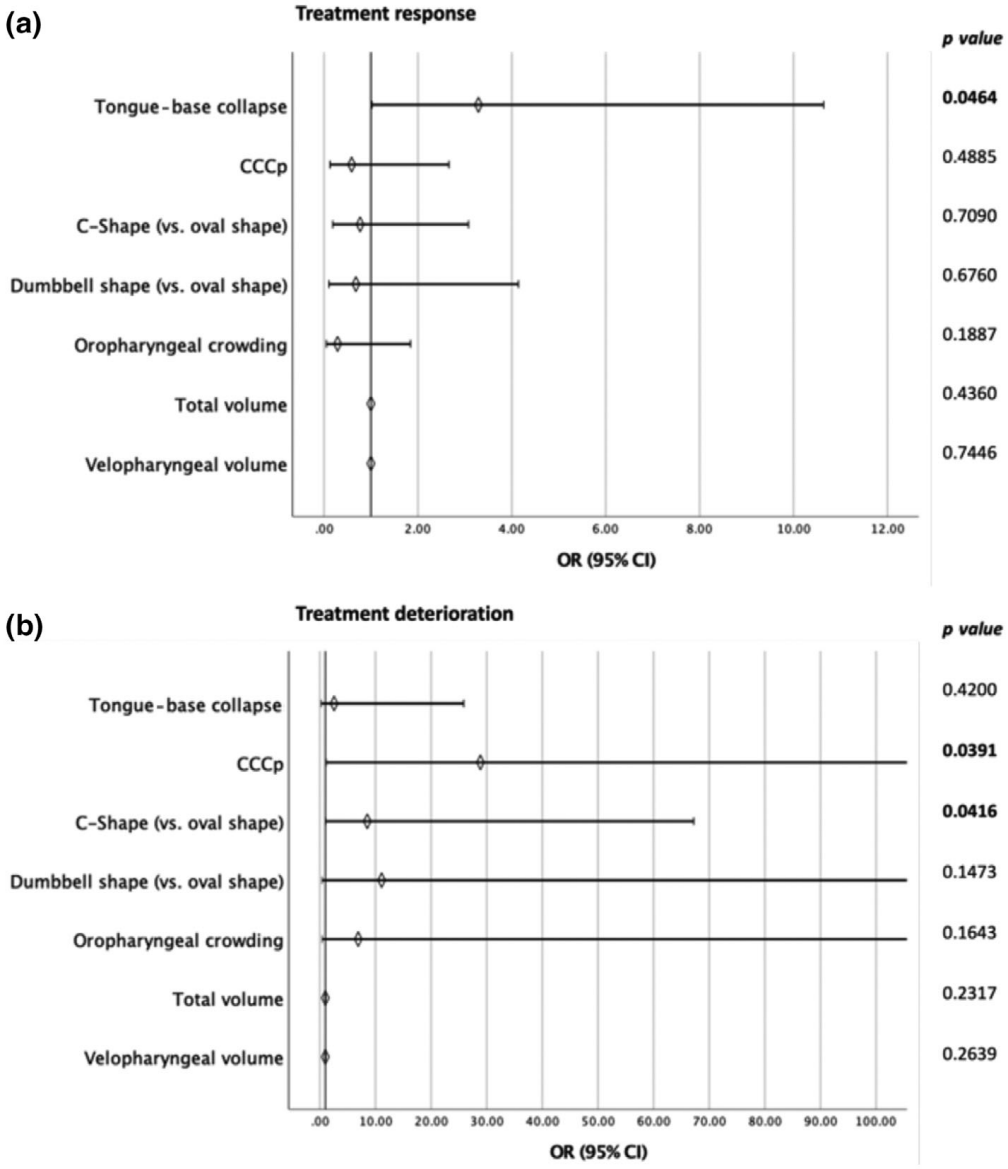
Outcome prediction for MAD treatment according to the different possible predictor variables using multivariate logistic regression. Prediction of response (a) and deterioration (b) of MAD treatment for each possible predictor variable using multivariate logistic regression combining all possible predictor variables in the imputed dataset and with correction of BMI and AHI. Significant values (*p* < 0.05) are shown in bold. Abbreviations: AHI, apnea–hypopnea index; BMI, body mass index; CCCp, complete concentric collapse at the level of the palate; CI, confidence interval; MAD, mandibular advancement device; OR, odds ratio

All previously identified potential predictor variables were combined, using multivariate logistic regression models according to MAD outcome, adjusted for BMI and baseline AHI (Table 7). The strongest impact concerning ORs was present for CCCp on deterioration (OR 28.88, 95% CI 1.18–704.35; *p* = 0.0391) (Figure 4b), followed by a C‐shape versus an oval shape of the soft palate on deterioration (OR 8.54, 95% CI 1.09–67.23; *p* = 0.0416), and tongue base collapse on response (OR 3.29, 95% CI 1.02–10.64; *p* = 0.0464) (Figure 4a). However, tongue base collapse remains the most stable predictor with a relatively narrow CI, after stepwise inclusion of all the predictive parameters in two separate multivariate models according to treatment outcome (Tables 8 and 9 **)**.

**TABLE 7:** Multivariate logistic regression according to mandibular advancement device outcome

	Response		Deterioration	
	*p*	OR (95% CI)	*p*	OR (95% CI)
DISE collapse				
Tongue base	**0.0464**	3.29 (1.02–10.64)	0.4200	2.58 (0.26–25.89)
CCCp	0.4885	0.59 (0.13–2.66)	**0.0391**	28.88 (1.18–704.35)
Awake nasendoscopy during tidal breathing				
Soft palate position				
‐ C‐shape (versus oval shape)	0.7090	0.77 (0.19–3.08)	0.0416	8.54 (1.09–67.23)
‐ Dumbbell shape (versus oval shape)	0.6760	0.68 (0.11–4.14)	0.1473	11.12 (0.42–293.21)
Oropharyngeal crowding	0.1887	0.29 (0.05–1.84)	0.1643	6.92 (0.45–106.50)
CFD				
Total upper airway volume	0.4360	1.00 (1.00–1.00)	0.2317	1.00 (1.00–1.00)
Velopharyngeal volume	0.7446	1.00 (1.00–1.00)	0.2639	1.00 (1.00–1.00)

Abbreviations: AHI, apnea–hypopnea index; CCCp, complete concentric collapse at the level of the palate; CFD, computational fluid dynamics; CI, confidence interval; DISE, drug‐induced sleep endoscopy; MAD, mandibular advancement device; OR, odds ratio.

*Note*: Data measured on imputed database. All regression analyses were adjusted for baseline AHI and body mass index.

**TABLE 8:** Stepwise multivariate logistic regression for response of mandibular advancement device treatment

DISE collapse	Response							
	+ AHI and BMI		+ AHI, BMI and awake nasendoscopy		+ AHI, BMI and CFD		+ AHI, BMI, awake nasendoscopy and CFD	
	*p*	OR (95% CI)	*p*	OR (95% CI)	*p*	OR (95% CI)	*p*	OR (95% CI)
Tongue base	**0.0294**	3.32 (1.13–9.75)	**0.0310**	3.57 (1.12–11.31)	**0.0449**	3.05 (1.03–9.04)	**0.0464**	3.29 (1.02–10.64)
CCCp	0.1987	0.41 (0.11–1.60)	0.3164	0.48 (0.12–2.01)	0.2764	0.45 (0.11–1.88)	0.4885	0.59 (0.13–2.66)

Abbreviations: AHI, apnea‐hypopnea index; BMI, body mass index; CCCp, complete concentric collapse at the level of the palate; CFD, computational fluid dynamics; CI, confidence interval; DISE, drug‐induced sleep endoscopy; MAD, mandibular advancement device; OR, odds ratio.

*Note*: Data measured on imputed database.

**TABLE 9:** Stepwise multivariate logistic regression for deterioration of mandibular advancement device treatment

DISE collapse	Deterioration							
	+ AHI and BMI		+ AHI, BMI and awake nasendoscopy		+ AHI, BMI and CFD		+ AHI, BMI, awake nasendoscopy and CFD	
	*p*	OR (95% CI)	*p*	OR (95% CI)	*p*	OR (95% CI)	*p*	OR (95% CI)
Tongue base	0.3585	1.99 (0.46–8.67	0.3703	2.37 (0.36–15.74)	0.5034	1.74 (0.34–9.00)	0.4200	2.58 (0.26–25.89)
CCCp	**0.0230**	6.25 (1.29–30.38)	**0.0359**	8.69 (1.15–65.58)	**0.0111**	23.35 (2.06–264.23)	**0.0391**	28.88 (1.18–704.35)

Abbreviations: AHI, apnea–hypopnea index; BMI, body mass index; CCCp, complete concentric collapse at the level of the palate; CFD, computational fluid dynamics; CI, confidence interval; DISE, drug‐induced sleep endoscopy; MAD, mandibular advancement device; OR, odds ratio.

*Note*: Data measured on imputed database.

### Diagnostic statistics and ROC analysis

**FIGURE 5:**
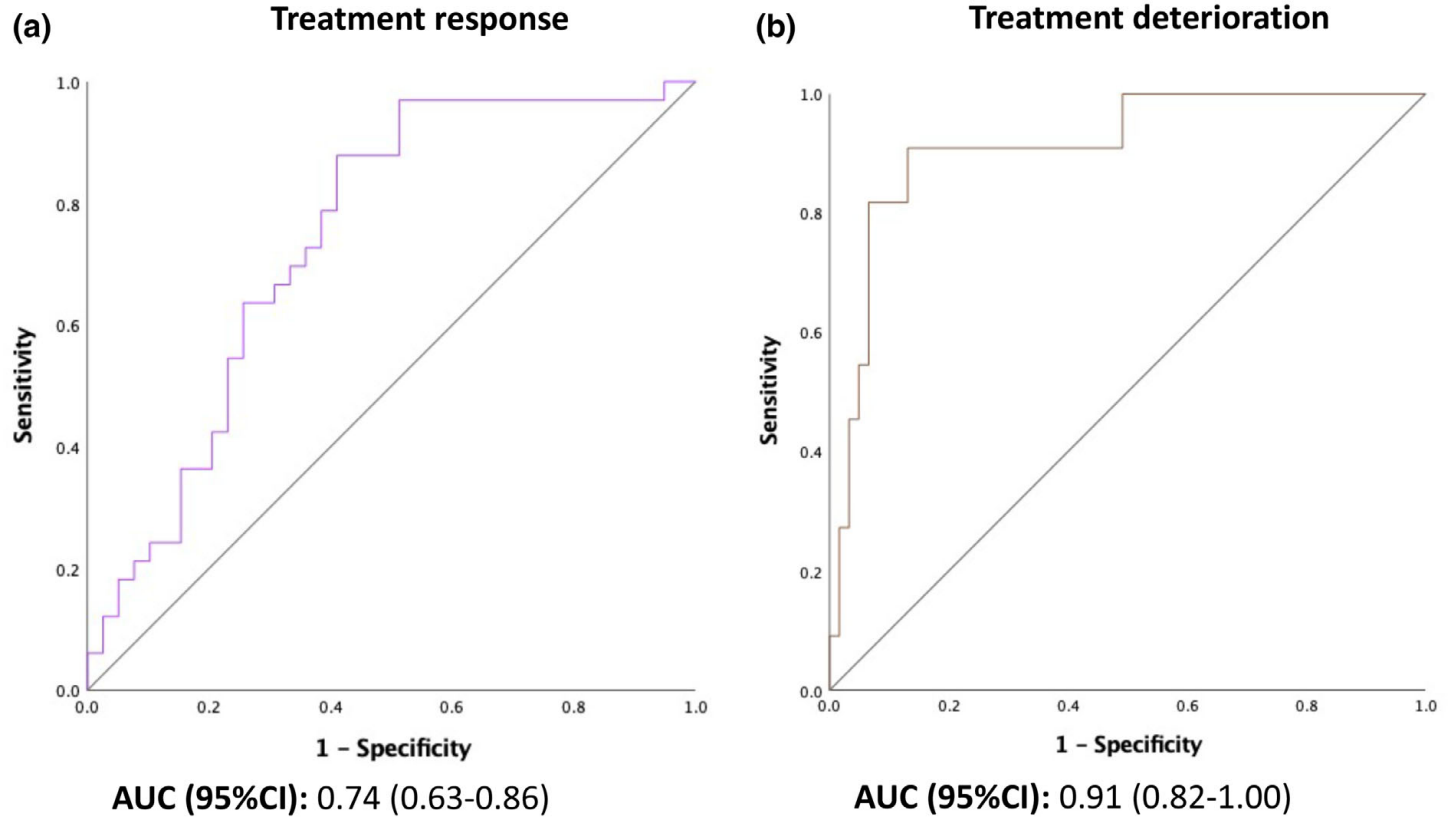
ROC curves for MAD outcome multivariate logistic regression prediction models. Separate prediction models were built for treatment response, defined as reduction in AHI of ≥50% and deterioration, defined as increase in AHI of ≥10% during MAD treatment. Abbreviations: AHI, apnea–hypopnea index; AUC, area under the curve; CI, confidence interval; ROC, receiver operating characteristic; MAD, mandibular advancement device

Diagnostic accuracy of both MAD outcome multimodal prediction models corrected for baseline AHI and BMI was determined, and ROC curves were generated (Figure 5). The optimal predictive probability cut‐off points, measured based on the maximal sensitivity and specificity using the Youden's index, were 0.24 for deterioration and 0.37 for response. The sensitivity/specificity for deterioration of MAD treatment was 90.9%/86.9% and for treatment response 87.9%/59.0%. A PPV of 60.8%, and a NPV of 97.8% with an area under the curve (AUC) of 0.91 could be obtained for the multivariate logistic model predicting treatment deterioration, derived from characteristics obtained during DISE, awake nasendoscopy, and CT scan‐based CFD, adjusted for baseline AHI and BMI. A PPV of 92.2%, and a NPV of 47.0% with an AUC of 0.74 could be retrieved from the model, predicting treatment response, adjusted for baseline AHI and BMI.

## DISCUSSION

In general, MAD treatment response is variable and patient dependent. Thus, careful patient selection is necessary to identify eligible patients and avoid unfavourable treatment, as this is associated with unnecessary costs and a longer delay toward successful treatment. Multiple studies here report several predictors for oral appliance therapy outcome, although multifactorial models and thorough validation are still lacking (Okuno et al., 2016).

Generally, an innovative clinical prediction model for MAD treatment outcome is outlined in this study, using patient characteristics obtained during DISE, awake nasendoscopy, and CT scan‐based CFD in patients with OSA.

The major findings of this prospective study suggest DISE to be the most robust examination associated with MAD treatment outcome, with tongue base collapse during baseline as a positive predictor for successful MAD treatment for OSA. Furthermore, the presence of CCCp is an adverse DISE phenotype towards MAD treatment outcome.

Firstly, the significant results of tongue base collapse regarding response and CCCp regarding deterioration during MAD treatment, are preserved using a multimodal assessment adjusted for awake nasendoscopy observations and CFD findings, and after correction for AHI and BMI. With this, a somewhat narrow CI is seen for tongue base collapse, enabling more precise interpretation of the results. CCCp presents a larger margin of error, necessitating a larger sample to adequately confirm these results, which emphasises tongue base collapse to be the most robust characteristic.

Regarding awake nasendoscopy, it is solely the presence of a prominent uvula (C‐shaped position of the soft palate) during tidal breathing that remains strongly correlated with MAD treatment deterioration after multimodal labelling. This contributes to the results of previous studies that a MAD primarily acts on the soft palate (Kent et al., 2015; Ryan et al., 1999).

Subsequently, no clear correlations have been found between MAD treatment outcome and baseline upper airway volume, as measured with CT scan‐based CFD. This is not completely surprising, as it is mainly the presence or absence of an increase in upper airway volume with the use of a MAD that seems to be significantly associated with treatment outcome in previous studies (Chan et al., 2010; Song et al., 2019; Van Gaver et al., 2021). However, other studies state that a narrower upper airway during both baseline awake examinations, including CT scan and awake nasendoscopy, as well as during DISE, are associated with a higher success rate of a MAD (Darquenne et al., 2018; Park et al., 2016; (Van de Perck, Op de Beeck, et al., 2020a). This emphasises the added value for combining this awake examination with investigations during drug‐induced sleep in our global model. Moreover, we presume that the combination of different predictor modalities may predominantly play an important role in outcome prediction, and that pharyngeal volume on its own is not exclusively associated with clinical MAD treatment outcome.

Moreover, both logistic regression models exhibit an excellent (AUC 0.8–0.9) and fair (AUC 0.7–0.8) predictive accuracy (Mandrekar, 2010). Implementation of these predictor variables may avoid treatment of patients with a lower probability of response or a high probability of deterioration with a MAD. However, at this point, these models are purely exploratory and are rather a representation of the presence or absence of their clinical applicability. Further validation of these results in a large cohort is thus needed.

### Strengths and limitations

All examinations were performed in a blinded fashion for the patient and multidisciplinary research team. Consequently, MAD treatment was not affected by the characteristics obtained during CT scan‐based CFD, DISE or awake nasendoscopy.

Although the various baseline prediction methods were performed at different timepoints, the timing only varied by up to 2 months between these examinations and between patients, minimising differences related to the time frame. Moreover, all examinations were performed at the earliest 1 month after MAD start, allowing a habituation period of 1 month.

Furthermore, both awake nasendoscopy and CT scans were performed during wakefulness, so the observed results may differ from a sleeping state, as changes in muscle tone at the upper airway occur predominantly during sleep (Fogel et al., 2004). However, the creation of a multimodal model with the addition of endoscopic examinations during drug‐induced sleep may overcome this problem and optimise the predictive value for MAD treatment outcome.

In this regard, treatment outcome was assessed using the difference in baseline AHI value and AHI with the use of a MAD, in which response was defined as a reduction in the AHI of ≥50% from baseline, as such pooling partial and complete responders. With this, a response rate of 45.8% was achieved, which is a similar to slightly lower result compared to other studies when using the same response criteria (Op de Beeck et al., 2020; Sutherland et al., 2015; Tsuiki et al., 2010; Van de Perck, Op de Beeck, et al., 2020a; Vroegop et al., 2013a). Furthermore, treatment deterioration was defined as an increase of ≥10% in the AHI with a MAD in comparison to baseline PSG measurements, resulting in 15.3% deterioration. This rather strict definition for treatment deterioration may represent a more uniform group in comparison with a negative response to MAD treatment ((Van de Perck, Op de Beeck, et al., 2020a).

Both measurements were only determined by a one‐night PSG, as such not considering internight variability. With this, postural changes, sleep structure, and a first night effect may also influence OSA severity (Sforza et al., 2019). However, in most studies, the proportion of patients who exhibit internight variability remains limited to between 18% and 35% (Alshaer et al., 2018; Bliwise et al., 1991). Therefore, the authors postulate that the effect of this limitation remains limited.

As standardised MAD‐titration guidelines are lacking, to create uniformity among the various patients, the MAD is fixed at 75% of maximal protrusion. In contrast, within clinical practice in our hospital, an optimal personalised titration is performed (generally ranging between 75% and 100% of maximal protrusion), which will probably improve treatment response. To objectify outcome prediction of MAD treatment here, a uniform fixed degree of protrusion was adopted within the present study.

Moreover, endoscopic examinations are rather subjective in nature and predisposed to high intra‐ and interobserver variability. In this regard, during previous DISE studies, a poor to good interobserver agreement has been observed (Kilavuz & Bayram, 2019; Vroegop et al., 2013b). Therefore, to avoid this constraint, a uniform classification system was implemented to score both awake nasendoscopic and DISE observations, in which all awake nasendoscopic findings were evaluated by the same ENT specialist and reviewed by a second experienced investigator. Furthermore, the DISE observations were scored during consensus scoring by four experienced ENT surgeons to reduce possible interobserver variability.

Lastly, a multiple imputation approach was implemented to deal with missing data, obtaining a complete dataset with approximately unbiased estimates of all potential predictive characteristics. This gives the advantage of performing further statistical analyses on a larger number of patients, which increases overall statistical power.

In conclusion, this is the first study to our knowledge, combining DISE findings and awake examinations in one model for predicting MAD treatment outcome. With this, a recent study has concluded that a prediction model with awake assessments has no added value in the prediction of MAD treatment outcome compared to the use of clinical baseline characteristics alone (Sutherland et al., 2018). However, our research highlights the necessity to find a robust clinical applicable prediction model, combining several predictors for optimising clinical decision‐making in routine practice for patients with OSA.

## CONCLUSION

It remains an ongoing challenge to predict the therapeutic effect of MAD treatment. A wide range of models combining clinical characteristics and different examinations awake and asleep have been introduced. However, thorough validation is lacking. Using multivariate logistic regression, combining characteristics obtained during DISE, awake nasendoscopy, and CT scan‐based CFD, our findings show that the combination of different predictor modalities may predominantly play an important role in outcome prediction of MAD treatment. Furthermore, the assessments during drug‐induced sleep appear to be more important compared to awake examinations. In particular, our results suggest DISE to be the most robust examination associated with MAD treatment outcome, with tongue base collapse during baseline being associated with a successful MAD treatment outcome and CCCp during baseline as an adverse DISE phenotype towards MAD treatment outcome.

## AUTHOR CONTRIBUTIONS

Sara Op de Beeck, Marijke Dieltjens, Olivier M. Vanderveken, Annelies E. Verbruggen, Anneclaire V. Vroegop, Johan A. Verbraecken, Paul H. Van de Heyning, Marc J. Braem worked on the conception. Sara Op de Beeck, Marijke Dieltjens, Olivier M. Vanderveken and Karlien Van den Bossche designed the methodology. Karlien Van den Bossche conducted the data analysis and drafted the final version of the article. All authors contributed to revision and final approval of the manuscript.

## CONFLICT OF INTEREST

Karlien Van den Bossche, Annelies E. Verbruggen, Anneclaire V. Vroegop and Paul H. Van de Heyning have no competing interests to declare. Sara Op de Beeck and Marijke Dieltjens hold a postdoctoral fellowship at the Research Foundation Flanders (FWO) – 1299822N and FWO – 12H4516N. Johan A. Verbraecken reports grants from SomnoMed outside the submitted work and sits on the advisory board of ResMed Narval. Marc J. Braem reports grants from SomnoMed outside the submitted work and sits on the advisory boards of ResMed and SomnoMed. Olivier M. Vanderveken reports grants from Philips, grants from SomnoMed, personal fees from SomnoMed, other from Inspire Medical Systems, personal fees from Inspire Medical Systems, other from Zephyr, other from Philips, outside the submitted work. Olivier M. Vanderveken holds a Senior Clinical Investigator Fellowship from the FWO – 1833517N.

## Data Availability

The data that support the findings of this study are available from the corresponding author upon reasonable request.
